# A sliding-window based algorithm to determine the presence of chest compressions from acceleration data

**DOI:** 10.1016/j.dib.2022.107973

**Published:** 2022-02-18

**Authors:** Wolfgang J. Kern, Simon Orlob, Birgitt Alpers, Michael Schörghuber, Andreas Bohn, Martin Holler, Jan-Thorsten Gräsner, Jan Wnent

**Affiliations:** a University of Graz, Institute of Mathematics and Scientific Computing, Heinrichstr. 36, Graz, Austria; b BioTechMed-Graz, Graz, Austria; c University Hospital Schleswig-Holstein, Institute for Emergency Medicine, Kiel, Germany; d Department of Anesthesiology and Intensive Care Medicine, Division of Anesthesiology for Cardiovascular and Thoracic Surgery and Intensive Care Medicine, Medical University of Graz, Graz, Austria; e Department of Anesthesiology, Intensive Care and Pain Medicine, University Hospital Münster, Münster, Germany; f City of Münster Fire Department, Münster, Germany; g Department of Anaesthesiology and Intensive Care Medicine, University Hospital Schleswig-Holstein, Kiel, Germany; h School of Medicine, University of Namibia, Windhoek, Namibia

**Keywords:** Cardiac arrest, Cardiopulmonary resuscitation, Chest compressions, Chest compression fraction, Accelerometry

## Abstract

This publication presents in detail five exemplary cases and the algorithm used in the article (Orlob et al. 2022). Defibrillator records for the five exemplary cases were obtained from the German Resuscitation Registry. They consist of accelerometry, electrocardiogram and capnography time series as well as defibrillation times, energies and impedance when recorded. For these cases, experienced physicians annotated time points of cardiac arrest and return of spontaneous circulation or termination of resuscitation attempts, as well as the beginning and ending of every single chest compression period in consensus, as described in Orlob et al. (2022). Furthermore, an algorithm was developed which reliably detects chest compression periods automatically without the time-consuming process of manual annotation. This algorithm allows for an usage in automatic resuscitation quality assessment, machine learning approaches, and handling of big amounts of data (Orlob et al. 2022).

**Specifications Table**

**Table:** 

Subject	Emergency medicine
Specific subject area	Cardiopulmonary resuscitation (CPR), CPR quality metrics, Chest compression fraction, Chest compression detection
Type of data	Supplemental files of continuous-time data in a repository Algorithm
How the data were acquired	Defibrillator records from adult resuscitation attempts, all ZOLL X-Series (ZOLL Medical Corporation, Chelmsford, Massachusetts, United States), Manual consensual annotations from experienced physicians with a web-based plotting tool using [2,3]
	Automatic computations of a newly developed algorithm.
Data format	Raw
	Analyzed
Description of data collection	Defibrillator recordings were prospectively collected and archived within the German Resuscitation Registry (GRR). Five exemplary defibrillator records were read out with Python based signal processing scripts. A web-based interactive plotting tool was used for the annotations by experienced emergency physicians. Dissenting annotations were re-assessed in consensus.
Data source location	Institution: German Resuscitation Registry
	City: Nürnberg
	Country: Germany
Data accessibility	Repository name: CPRDAT 0.1
	Data identification number: DOI: 10.5281/zenodo.5497699
	Direct URL to data: https://doi.org/10.5281/zenodo.5497699
Related research article	S. Orlob, W. J. Kern, B. Alpers, M. Schörghuber, A. Bohn, M.Holler, J.-T. Gräsner, J. Wnent, Chest compression fraction calculation: Chest compression fraction calculation: A new, automated, robust method to identify periods of chest compressions from defibrillator data - Tested in Zoll X Series., Resuscitation, In Press. [1]

**Value of the Data**

• These data are useful since they contain multichannel continuous defibrillator recordings as well as consensual annotations from experienced physicians, which allow a full description and characterization of a CPR case. Furthermore, a highly reliable algorithm to determine the presence of chest compressions directly from accelerometer data without any manual annotations is provided.
• Researchers working on cardiac arrest treatment and its quality metrics benefit from this new method to detect chest compression periods automatically, since the highly time-consuming, manual annotation of single chest compressions can be avoided [4]. Thus, the new method allows for widespread usage including post-event debriefing, CPR-metric-calculations in large registries [5], and machine learning approaches.
• The present data and the presented algorithm can be used for further research in this field, providing a reliable automatic determination of the presence of chest compressions. Beside from easily computing CPR-metrics, a reliable, automatic chest compression period detection is a basic, but crucial tool for future research in this field using machine learning algorithms and big amounts of data.
• The present data and the presented algorithm allow the computation of standardized CPR quality metrics [6] like Chest Compression Fraction (CCF) with much less effort compared to conventional workflows that rely on manual annotation of single chest compressions, thereby allowing for a widespread usage of these measures to assess CPR quality.
• This algorithm facilitates the computation of CCF in clinical quality management of emergency response systems as well as in clinical research as quality monitoring tool, providing feedback to emergency medical services to improve their performance in CPR.
• Even though this algorithm was developed only for acceleration data of one defibrillator manufacturer, we expect the general principle to be applicable to other manufacturers and to thoracic impedance as well. Its open source nature and its generic structure allows for an incorporation into general CPR analysis frameworks [7].

## 1 Data Description

The data consists of two different data types. Five exemplary recordings from the German Resuscitation Registry are provided in https://doi.org/10.5281/zenodo.5497699 in the subfolder cc-periods_cases. Each case is saved in its own directory which contains several csv-files. The recording of each case consists of time series of acceleration measurement, electrocardiogram from the shock electrodes and capnography. Detailed information about these channels can be found in Table 1. All time data is given in seconds that have elapsed since the defibrillator was switched on.

**Table 1:** Units and sample rates of all provided continuous-time data.

Channel	Accelerometer	Shock electrodes	Capnography
Description	acceleration of chest during the whole recording	electrocardiogram recorded by the shock electrodes	sidestream capnography, continuous time expiratory CO_2_ concentration
**Unit**	internal units	mV	mmHg
**Sample rate / Hz**	250	250	125
**File name**	Accelerometer.csv	ShockElectrodes.csv	Capnography.csv

Additionally, the following side-data is provided: time and energy [J] of defibrillation shocks, thoracic impedance [Ω] while defibrillation shocks, consensual annotations (Arrest and Return of spontaneous circulation (ROSC) / CPR-termination timepoints, Start and Stop markers for each chest compression period) by two experienced physicians, Start and stop markers for each chest compression period derived from the new algorithm. Details about the annotations can be found in Table 2.

**Table 2:** Description of all manual annotations.

Annotation	Description	File name
Arrest	Supposed time of the cardiac arrest / rearrest	PhysioStatus.csv
ROSC / Termination	Supposed time of the ROSC or termination of CPR without ROSC	PhysioStatus.csv
CC-period-start	Annotated start time of a single chest compression period	Ann_CC-periods.csv
CC-period-end	Annotated stop time of a single chest compression period	Ann_CC-periods.csv

The terms “Arrest” and “ROSC/Termination” describe the physiological state of the patient, where performing CPR is compulsory between “Arrest” and “ROSC/Termination”. The timepoint annotated with “ROSC/Termination” can either mark an occurring ROSC or the termination of CPR, when the resuscitation attempt fails. Additionally, the algorithm described below allows an automatic computation of the start and stop markers of single chest compression periods for each case. These start and stop markers can be compared to the manual annotations. These markers computed by the algorithm are given in Alg_CC-periods.csv. The algorithm can be executed directly by running the Jupyter-notebook CC-periods.ipynb, which is also part of the repository mentioned above.

## 2 Experimental Design, Materials and Methods

All the raw data was recorded by ZOLL X-Series - (ZOLL Medical Corporation, Chelmsford, Massachusetts, United States) and have been stored in the German Resuscitation Registry. The data have been processed in Python (Python v.3.7.3). For the manual annotations an interactive, web-based plotting tool [2], [3] was used by experienced emergency physicians. Each case was annotated by two independent physicians and annotations deviating more than 0.5 s were reset in consensus.

**Fig. 1:**
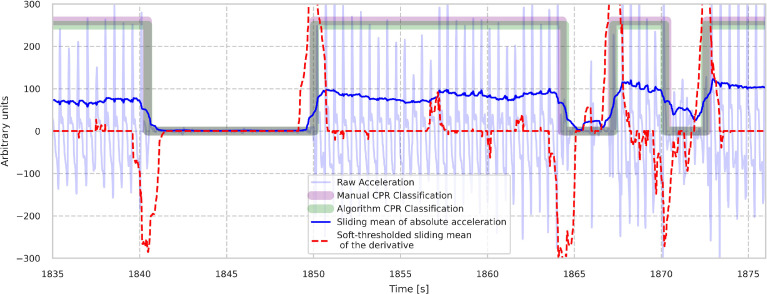
Illustration of the algorithm work-flow. Data from Case_1. The raw acceleration data is given by the pale blue line, while the sliding mean of the absolute acceleration is shown in solid blue. The soft shrunk sliding average of the first derivative of the solid blue line is shown in dashed red. The maxima and minima of this quantity are the candidates for the beginnings and endings of the single CC-sets. The resulting classification of the algorithm (green) as well of the manual annotations (purple) are shown as rectangular functions. (chest compressions absent ⋯=0, chest compressions present ⋯≠0).

The algorithm computes the start and end times of single chest compression periods directly from the accelerometer data. The process described successively is illustrated in Fig. 1. First, the acceleration data gets broadly bandpass-filtered with a fourth order Butterworth filter and critical frequencies of 0.2 Hz and 50 Hz, respectively, in order to remove slow baseline changes of the accelerometer and the stationary contributions from gravitation acceleration as well as high frequency noise. Then a centered sliding mean over a 1 s interval of the absolute mean of the acceleration is computed. This quantity is nearly constant and non-vanishing while compressions are present and is approximately vanishing and constant during pauses. The algorithm computes the average mean of the first derivative of this quantity and performs a soft shrinkage step to remove little, non-significant minima and maxima of the first derivative.

Afterwards, the algorithm searches alternately for the absolute largest extremum of one kind (e.g. maximum) between extrema of the other kind (e.g. minima) or conversely. These positions of alternating maxima and minima are the candidates for the start and end times of the chest compression periods (see Fig. 1), and with that, define potential chest compression periods with pauses in between.

**Fig. 2:**
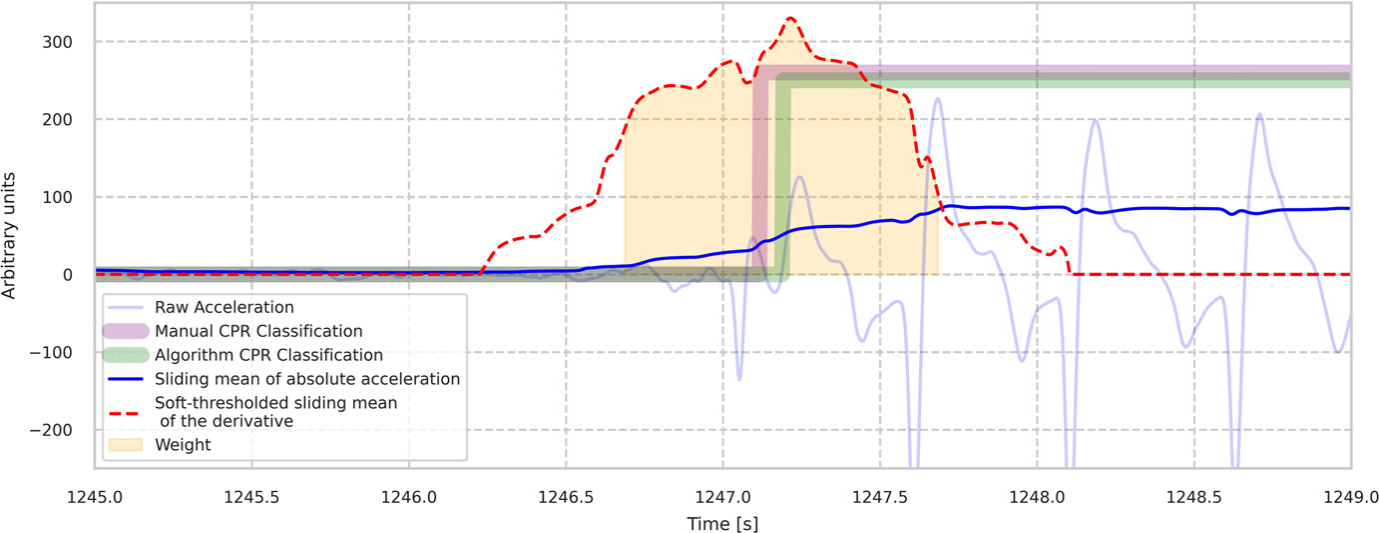
Illustration of the effect of the weigthed mean. Data from Case_1. Due to the oscillatory character of the acceleration (pale blue), the derivative of the sliding mean of the absolute acceleration exhibits two large maxima (red dashed line). The algorithm corrects the start of the chest compression period by taking a weighted mean with the absolute of the sliding mean of the derivative as a weight (orange area). The predicted start of chest compression is then moved slightly to the left from the global maximum towards the annotated start point.

In a next step, the algorithm filters these candidates by requiring the following three conditions: First, the mean of the absolute acceleration during a pause must be less than 35% of the average of the two means of the absolute acceleration of the chest compression periods before and after. Second, a pause must last for at least 1.6 s. Third, during the corresponding chest compression periods the mean of the absolute acceleration must not be below a certain threshold value. Finally, if two similar large maxima of the absolute derivative are found, in order to further improve the position of marker, the start/stop marker is set in between these maxima by using a weighted mean. The weight is given by a one second long interval of the absolute derivative centered around the maximum. (see Fig. 2).

## Ethics Statements

This study was approved by the ethics committee of the University of Kiel (Ref. no.: D 421/21) and the scientific advisory board of the German Resuscitation Registry (Ref. no.: AZ 2021-03)

## CRediT authorship contribution statement

**Wolfgang J. Kern:** Conceptualization, Methodology, Software, Validation, Formal analysis, Writing – original draft, Writing – review & editing, Visualization. **Simon Orlob:** Conceptualization, Methodology, Software, Validation, Investigation, Data curation, Writing – review & editing. **Birgitt Alpers:** Data curation, Writing – review & editing. **Michael Schörghuber:** Data curation, Writing – review & editing. **Andreas Bohn:** Investigation, Resources, Writing – review & editing. **Martin Holler:** Conceptualization, Methodology, Formal analysis, Writing – original draft, Supervision, Project administration, Funding acquisition. **Jan-Thorsten Gräsner:** Resources, Writing – review & editing, Supervision, Funding acquisition. **Jan Wnent:** Conceptualization, Investigation, Data curation, Writing – review & editing, Supervision, Project administration.

## Declaration of Competing Interest

JTG has received speakers honorary from ZOLL Medical, not related to the content of this study. All other authors have no conflict of interest to declare.
